# SGLT2 inhibitors for patients with type 2 diabetes mellitus after myocardial infarction: a nationwide observation registry study from SWEDEHEART

**DOI:** 10.1016/j.lanepe.2024.101032

**Published:** 2024-08-21

**Authors:** Hans Christian Rosén, Moman A. Mohammad, Tomas Jernberg, Stefan James, Jonas Oldgren, David Erlinge

**Affiliations:** a Department of Cardiology, Clinical Sciences, Lund University, Skåne University Hospital, Lund, Sweden; b Department of Clinical Sciences, Danderyd, Hospital, Karolinska Institutet, Stockholm, Sweden; c Uppsala Clinical Research Center, Uppsala University, Uppsala, Sweden; d Department of Medical Sciences, Cardiology, Uppsala University, Uppsala, Sweden

**Keywords:** Myocardial infarction, Sodium-glucose co-transporter-2 (SGLT2) inhibitors, Type 2 diabetes mellitus, Heart failure, SWEDEHEART

## Abstract

**Background:**

Sodium-glucose co-transporter 2 (SGLT2) inhibitors have been shown to reduce rates of heart failure hospitalisations and cardiovascular death in patients with type 2 diabetes and prior cardiovascular disease. We hypothesised that SGLT2 inhibitors could provide cardiovascular benefits in the post-myocardial infarction setting. We aimed to investigate cardiovascular outcomes of SGLT2 inhibitor therapy in patients with type 2 diabetes mellitus after myocardial infarction in a Swedish nationwide registry.

**Methods:**

We included all patients with type 2 diabetes surviving a type 1 acute myocardial infarction from January 1, 2018 to December 31, 2021. Patients were included if they were discharged with an estimated glomerular filtration rate (eGFR) > 30 mL/min/1.73 m^2^ in the nationwide Swedish Web-system for Enhancement and Development of Evidence-based care in Heart disease Evaluated According to Recommended Therapies (SWEDEHEART) registry. We identified all patients discharged with or without an SGLT2 inhibitor prescription 120 days before or within three days after discharge from the cardiac care unit. The primary outcome measure was a composite of death and first hospitalisation for heart failure after one year analysed using an adjusted Cox regression.

**Findings:**

A total of 11,271 patients were included. Of these, 2498 (22.2%) received SGLT2 inhibitor treatment. Patients who were prescribed SGLT2 inhibitors were younger, more often presented with a STEMI and had worse left ventricular ejection fraction at index hospitalisation. SGLT2 inhibitor use was associated with lower rates of the composite outcome (hazard ratio (HR) of 0.70 (95% confidence interval (CI) 0.59–0.82).

**Interpretation:**

Treatment with SGLT2 inhibitors after myocardial infarction in patients with type 2 diabetes was associated with a lower rate of cardiovascular events.

**Funding:**

This work was supported by 10.13039/501100003793Hjärt-Lungfonden, Vetenskapsrådet, Knut and Alice Wallenberg Foundation, ALF, the 10.13039/501100021728Bundy Academy, and Skåne University Hospital funds.

**Research in context**

**Evidence before this study**

SGLT2 inhibitors have proven cardioprotective properties in patients with type 2 diabetes, especially among individuals with a high cardiovascular risk profile. We searched PubMed to find studies on the use of SGLT2 inhibitors in patients with type 2 diabetes in the post-myocardial infarction phase using the terms “myocardial infarction”, “diabetes mellitus”, in addition to “SGLT2-inhibitor”, “sodium-glucose cotransporter 2 inhibitor”, “canagliflozin”, “empagliflozin”, and “dapagliflozin” from Jan 1, 2010 to Dec 1, 2023. Very few studies included patients with type 2 diabetes treated with SGLT2 inhibitors following a myocardial infarction. One randomised trial reported improved laboratory values of heart failure and echocardiographic parameters but did not report on cardiovascular endpoints. One smaller registry trial reported lower rates of MACE. One observational, single-centre trial of patients with type 2 diabetes and a recently diagnosed extensive coronary artery disease reported a reduction of all-cause mortality.

**Added value of this study**

We conducted a nationwide observational registry study to analyse any effect on cardiovascular outcomes in patients with type 2 diabetes treated with an SGTL2 inhibitor following a myocardial infarction in Sweden. We found evidence of an association between lower rates of all-cause death and first hospitalisations of heart failure among SGLT2 inhibitor-treated patients following a myocardial infarction.

**Implications of all the available evidence**

These findings suggest that treatment might lower cardiovascular events among patients with type 2 diabetes, even in the early post-myocardial infarction phase. Evidence from randomised controlled trials and long-term safety data will elucidate the potential benefits from treatment further.

## Introduction

The link between type 2 diabetes and coronary artery disease is well-established, and those with diabetes who survive a myocardial infarction are at high risk of developing new cardiovascular events, including the development of heart failure and death.^1^^,^^2^ Sodium-glucose co-transporter 2 (SGLT2) inhibitors have demonstrated reductions of cardiovascular and renal adverse events in several trials and are today part of established treatment practices for type 2 diabetes mellitus, chronic kidney disease (CKD), and heart failure.^3^, ^4^, ^5^ SGLT2 inhibitor treatment has been shown to reduce the risk of hospitalisation for heart failure, cardiovascular mortality, and major adverse cardiac events (MACE) in patients with type 2 diabetes and high risk for or established cardiovascular disease.^6^, ^7^, ^8^, ^9^, ^10^ Patients with a recent myocardial infarction were excluded from these trials which prompted the DAPA-MI and EMPACT-MI trials to explore early SGLT2 inhibitor treatment in patients surviving a myocardial infarction.^11^^,^^12^ SGLT2 inhibitor treatment in DAPA-MI showed a significant reduction in cardiometabolic outcomes, primarily driven by a lowered risk of developing diabetes and reductions in body weight. EMPACT-MI did not show a significant reduction of the primary endpoint of all-cause death and first hospitalisation for heart failure. However, the secondary analysis demonstrated a significant reduction in first hospitalisations for heart failure. We hypothesised that the cardiovascular benefits associated with SGLT2 inhibitor treatment could be observed in patients with type 2 diabetes after a myocardial infarction. The DAPA-MI trial excluded all patients with known type 2 diabetes, and only 32% of patients in the EMPACT-MI had type 2 diabetes. In this study, we aimed to study the risk of hospitalisations for heart failure and mortality in patients with type 2 diabetes treated with SGLT2 inhibitor after a myocardial infarction.

## Methods

### Data sources

In this study, we utilised data from the national quality registries linked to the Swedish Web-system for Enhancement and Development of Evidence-based care in Heart disease Evaluated According to Recommended Therapies (SWEDEHEART) registry. The purpose and organisation of the registry have been previously described in detail.^13^ In short, the SWEDEHEART registry is a nationwide registry collecting information on patient characteristics and procedural information related to acute and chronic cardiac diseases. Patients from all hospitals with acute cardiac care in Sweden (n = 72) are registered and notified of their inclusion in the registry and their right to opt out. Patients are organised by their unique personal identification number, which is issued to all residents in Sweden. In the present study, patient baseline characteristics such as previous diagnosis, risk factors, and medical therapy were retrieved from the SWEDEHEART registry. Using the SWEDEHEART registry, patients can be cross-referenced by their personal identification number with other Swedish registries, a practice approved by the National Board of Health and Welfare. The SWEDEHEART registry was cross-referenced to the National Prescribed Drug Register for details on the redemption of SGLT2 inhibitors, to the National Patient Register for information on stroke and hospitalisations of heart failure, and to the Swedish Population Registry for information on the vital status. This study has been approved by the Ethics Review Board in Lund (approval id 2015/297) and was conducted in accordance with the principles of the Declaration of Helsinki.

### Study population

We included all adults (≥18 years) with established type 2 diabetes discharged from the cardiac care unit in Sweden following a type 1 myocardial infarction between January 1, 2018, and December 31, 2021. The diagnosis of myocardial infarction was determined by the treating physician according to the current definition of myocardial infarction.^14^ For patients with multiple entries in the SWEDEHEART registry, the most recent hospitalisation was used as the index event. Patient inclusion and exclusion criteria can be found in the flowchart in Supplemental Figure S1. Type 2 diabetes was defined according to the International Statistical Classification of Diseases and Related Health Problems, 10th revision (ICD-10) codes (E110-119), or treated diabetes upon presentation, or discharge with a diabetes diagnosis according to the diagnostic criteria by the WHO. Patients with an estimated glomerular filtration rate (eGFR) ≤ 30 mL/min/1.73 m^2^ according to the CKD-EPI formula were excluded as SGLT2 inhibitors were contraindicated. Patients were considered to be on treatment with SGLT2 if they redeemed a prescription of SGLT2 inhibitor (ATC codes A10BK01–07, A10BD15,16,19-21,23-25,27) within 120 days before hospital discharge or were prescribed SGLT2 inhibitor during hospitalisation or redeemed prescription within three days.

### Outcomes

The primary outcome was the incidence of a composite of all-cause death and first hospitalisation for heart failure. The secondary outcomes were first hospitalisation for heart failure, all-cause death, and stroke considered separately. Follow-up time started at the time of discharge from the cardiac care unit and continued for one year. Information on patient vital status and date of death were obtained from the Swedish population registry. Hospitalisation for heart failure was defined as a registered hospitalisation with heart failure as the primary diagnosis (ICD-10, I50) following discharge as determined by the treating physician. Stroke was defined as a registered hospitalisation with cerebral stroke (ICD-10, I63) as the primary diagnosis as determined by the treating physician.

### Statistical analysis

#### Continuous variables and categorical variables

Baseline characteristics are presented as proportions for categorical variables and medians with interquartile ranges. Kaplan–Meier estimators and associated cumulative event rates were calculated. The proportional hazards assumption was checked using visual assessments of -ln-ln Kaplan–Meier survival curves and adjusted survival curves in addition to a goodness-of-fit test. A multivariable Cox regression model was used to calculate hazard ratios (HR) with an associated 95% CI for the primary and secondary outcomes using imputed values. Variables used to adjust the model were chosen based on previous knowledge and clinical relevance. The potential confounder variables used to adjust the imputed Cox regression were patient baseline characteristics (age, sex, body mass index, estimated glomerular filtration rate at index hospitalisation, HbA1c-level at index hospitalisation, smoking status, type of myocardial infarction (STEMI or NSTEMI), Killip class at index hospitalisation, left ventricular ejection fraction at index hospitalisation, and year of hospitalisation for myocardial infarction, Clinical Frailty Scale-score), comorbidities (previously or newly diagnosed hypertension, previous diagnosis of chronic obstructive pulmonary disease, stroke, heart failure, or myocardial infarction), and medical treatment at discharge from the coronary care unit (statins, angiotensin-converting-enzyme inhibitors (ACEi) or angiotensin 2-receptor blockers (ARB), aspirin, beta-blockers, and insulin treatment). Multiple imputation by chained reaction was performed to account for missing values (number of iterations = 10) to impute variables with missing values (hypertension, prior myocardial infarction, smoking status, left ventricular function, Killip Class, Clinical Frailty Scale, estimated GFR, BMI, HbA1c, treatment with insulin, beta-blockers, aspirin, and ACEi/ARB) using the following predictors: age, sex, year of myocardial infarction, infarct type, prior diagnosis of; heart failure, chronic obstructive pulmonary disease, or stroke, SGLT2 inhibitor treatment and if outcomes occurred during the follow-up. Invasive coronary angiography and PCI during hospitalisation were added as potential confounder variables in one sensitivity analysis; while potential angiographical confounders (number of diseased vessels, the presence of proximal or distal disease and if bifurcation PCI was conducted) were added in an additional sensitivity analysis. A sensitivity analysis using an inverse probability-weighted Cox regression was performed. This analysis was conducted using the same variables except for HbA1c and Clinical Frailty Scale-score which were excluded due to the high proportion of missing data. A complete case sensitivity analysis was conducted using a multivariable logistic regression model with the same adjustment variables as in the primary analysis. A sensitivity analysis in patients with a low risk of heart failure was conducted in the subpopulation with LVEF ≥50% and no prior heart failure. A subgroup analysis of the composite outcome was examined in men vs women, age ≥75 vs < 75 years, estimated glomerular filtration rate ≥60 mL/min/1.73 m^2^ vs < 60 mL/min/1.73 m^2^, left ventricular ejection fraction at index hospitalisation, BMI ≥30 vs < 30 kg/m^2^, prior myocardial infarction vs no prior myocardial infarction, and year of index myocardial infarction 2018 vs 2019 vs 2020 vs 2021, and classification of myocardial infarction according to STEMI vs NSTEMI. Myocardial infarction classification was also studied Results from this analysis are presented using Cox regression and p-values for interaction. The statistical analyses were performed in Stata version 18.0 (StataCorp LLC). A two-sided p < 0.05 was considered statistically significant.

### Role of the funding source

The funders of the study had no role in data collection, study design, data analysis, data interpretation, writing or decision to publish the manuscript.

## Results

### Patient characteristics

A total of 11,271 patients with type 2 diabetes mellitus were discharged from the cardiac care unit following a type 1 myocardial infarction and included in this study (Table 1). Of these, 2498 (22.2%) were treated with an SGLT2 inhibitor. Patients treated with SGTL2 inhibitors were generally younger, more likely to be male or smokers, had a higher eGFR, were more often treated due to an ST-elevation myocardial infarction (STEMI), had previously suffered from myocardial infarction and undergone percutaneous coronary intervention (PCI), more likely to have hyperlipidaemia, and had worse left ventricular ejection fraction. Patients treated with SGLT2 inhibitors were more often treated with statins, angiotensin-converting enzyme inhibitors, or angiotensin receptor blockers. Patients not treated with an SGLT2 inhibitor had more often been diagnosed with cancer, stroke, chronic obstructive pulmonary disease, and peripheral vascular disease. Patients treated with SGLT2 inhibitors were more likely to have undergone coronary angiography and PCI during the index hospitalisation (Supplemental Table S1). There was no significant difference in the number of diseased vessels, prevalence of proximal coronary artery disease, or bifurcation PCI rates between the two groups.

**Table 1:** Patient characteristics at discharge by treatment with SGLT2 inhibitors.

	SGLT2 inhibitor	No SGLT2 inhibitor	Missing	p-value
Total population	2498 (22.2%)	8773 (77.8%)	–	–
Follow up time, days (median, IQR)	349 (139–649)	726 (333–1078)	–	–
Age (median, IQR)	69 (61–75)	73 (65–79)	0.0%	<0.001
Women	554 (22.2%)	2689 (30.7%)	0.0%	<0.001
SGLT2 inhibitor prior to admission	766 (39.0%)	–	21.3%	–
Smoking			4.4%	0.001
Active smoker	515 (21.5%)	1533 (18.3%)		
Previous smoker	984 (41.1%)	3711 (44.3%)		
Never smoker	898 (37.5%)	3138 (37.4%)		
BMI (IQR)	28 (26–32)	28 (25–32)	3.4%	0.049
eGFR ml/min/1.73 m^2^ (IQR)	83 (65–95)	76 (56–91)	0.3%	<0.001
HbA1c (median, IQR)	57 (48–70)	54 (47–65)	50.2%	<0.001
Diagnosis			0.0%	<0.001
STEMI	969 (38.8%)	2671 (30.5%)		
NSTEMI	1529 (61.2%)	6101 (69.6%)		
Coronary angiography	2411 (96.5)	7905 (90.1)	0.0%	<0.001
PCI	2074 (83.0%)	6532 (74.5%)	0.0%	<0.001
Killip class			2.6%	0.003
1	2214 (90.6)	7591 (89.0)		
2	161 (6.6)	737 (8.6)		
3	38 (1.6)	131 (1.5)		
4	31 (1.3)	73 (0.9)		
LVEF			10.5%	<0.001
≥50%	1139 (49.3%)	4505 (57.9%)		
40–49%	581 (25.1%)	1833 (23.6%)		
30–39%	408 (17.7%)	997 (12.8%)		
<30%	184 (8.0%)	440 (5.7%)		
Clinical frailty score (median, IQR)	3 (2–3)	3 (2–3)	28.6%	0.005
Previous MI	923 (37.1%)	2989 (34.2%)	0.4%	0.016
Previous PCI	828 (33.2%)	2535 (28.9%)	0.4%	0.002
Previous CABG	247 (9.9%)	841 (9.6%)	0.0%	0.65
Prior heart failure	287 (11.5%)	1124 (12.8%)	0.0%	0.078
Prior cancer diagnosis	86 (3.4%)	415 (4.7%)	0.0%	0.006
Hypertension	2008 (80.5%)	7148 (81.7%)	0.2%	0.18
Hyperlipidaemia	1442 (57.8%)	4875 (55.6%)	0.1%	0.013
Stroke	200 (8.0%)	914 (10.4%)	0.0%	<0.001
COPD	158 (6.3%)	788 (9.0%)	0.0%	<0.001
PVD	158 (6.3%)	647 (7.4%)	0.0%	<0.001
Medications at discharge				
ACEi/ARB	2213 (88.6%)	7579 (86.4%)	0.0%	0.004
Beta-blocker	2102 (84.2%)	7243 (82.6%)	0.0%	0.063
Aspirin	2179 (87.2%)	7645 (87.1%)	0.0%	0.90
Statin	2397 (96.0%)	8154 (92.9%)	0.1%	<0.001
Insulin	912 (36.5%)	3145 (35.9%)	0.0%	0.55
Metformin	1870 (74.9%)	6672 (76.1%)	0.0%	0.22
GLP-1 receptor agonist	327 (13.1%)	886 (10.1%)	0.0%	<0.001
ICD implantation at follow-up	18 (1.9%)	27 (1.0%)	0.0%	0.024
SGLT2 inhibitors			2.4%	–
Dapagliflozin	480 (19.2%)	–		
Empagliflozin	1951 (78.1%)	–		
Canagliflozin	8 (0.3%)	–		
Year of index myocardial infarction			0.0%	<0.001
2018	201 (8.1%)	2463 (28.0%)		
2019	281 (11.3%)	2471 (28.2%)		
2020	729 (29.2%)	2022 (23.1%)		
2021	1287 (51.5%)	1817 (20.7%)		

ACEi, Angiotensin-converting enzyme inhibitors; ARB, Angiotensin II receptor blockers; BMI, Body mass index; CABG, Coronary artery bypass grafting; COPD, chronic obstructive pulmonary disease; eGFR, Estimated glomerular filtration rate; GLP-1, glucagon-like peptide 1; ICD, implantable cardioverter defibrillator; IQR, Interquartile range; MI, Myocardial infarction; NSTEMI, Non-ST-elevation myocardial infarction; PCI, percutaneous coronary intervention; PVD, Peripheral vascular disease; SGLT2, Sodium-glucose co-transporter 2; STEMI, ST-elevation myocardial infarction.

### Primary outcome

**Fig. 1:**
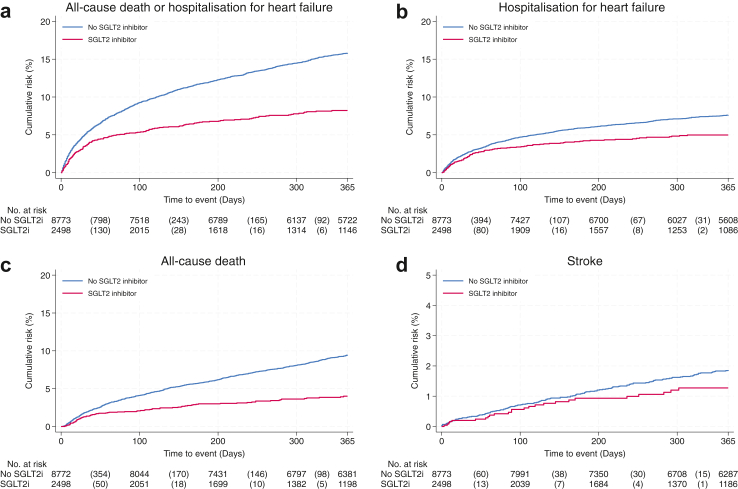
**Cumulative incidence of all-cause death or hospitalisation for heart failure (Panel a), hospitalisation for heart failure (Panel b), all-cause death (Panel c) and stroke (Panel d)**.

The rates of the primary outcome of a composite of first hospitalisations for heart failure or all-cause death are presented in Fig. 1. The primary outcome occurred in 1479 patients during the 365 days of follow-up. The Kaplan–Meier rate of the primary outcome was lower (8.2 vs. 15.8) among those treated with an SGLT2 inhibitor than those not treated with an SGLT2 inhibitor (Table 2). After multivariable adjustment, SGLT2 inhibitor treatment was associated with a 30% lower hazard of the composite outcome (HR 0.70; 95% CI, [0.59–0.82]) (Fig. 1).

**Table 2:** Results of the primary and secondary outcome analyses with one-year follow-up.

	Number of events among SGLT2 inhibitor treated	Number of events among non-SGLT2 inhibitor treated	KM-rate among SGLT2 inhibitor treated	KM-rate among non SGLT2 inhibitor treated	Unadjusted HR (95% CI)	Adjusted HR (95% CI)
Composite of first hospitalisation for heart failure or all-cause death	180	1299	8.2	15.8	0.52 (0.45–0.61)	0.70 (0.59–0.82)
First hospitalisation for heart failure	106	600	5.0	7.6	0.66 (0.54–0.82)	0.78 (0.63–0.98)
All-cause death	83	772	4.0	9.5	0.43 (0.35–0.54)	0.64 (0.50–0.80)
Stroke	25	144	1.3	1.9	1.12 (0.63–1.97)	0.86 (0.54–1.35)

Adjusted for age, sex, eGFR, BMI, HbA1c-level, smoking status, clinical frailty score, Killip class at admission, MI classification (STEMI/NSTEMI), left ventricular ejection fraction, year of admission, treatment with angiotensin-converting enzyme inhibitors/angiotensin II receptor blockers, beta-blockers, statins, aspirin and insulin, diagnosis of hypertension and COPD, and prior stroke, heart failure, and myocardial infarction.

CI, Confidence interval; HR, Hazard ratio; KM, Kaplan–Meier.

### Secondary outcome

**Fig. 2:**
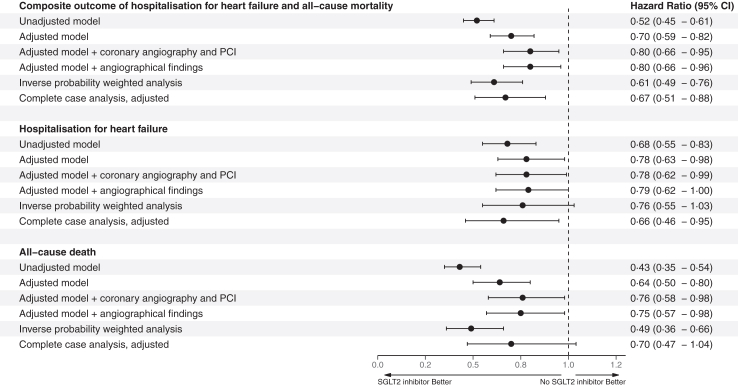
Results of the primary and secondary outcome analyses with one-year follow-up.

The Kaplan–Meier rates of first hospitalisation for heart failure, all-cause death and stroke are presented in Fig. 1. The Kaplan–Meier rates were lower in those treated with SGLT2 inhibitors for both first hospitalisation for heart failure (5.0 vs 7.6), for all-cause death (4.0 vs 9.5) and stroke (1.3 vs 1.9) (Table 2). The multivariable-adjusted hazard ratios were lower for first hospitalisation for heart failure (HR 0.78; 95% CI [0.63–0.98]), for all-cause death (HR 0.64; 95% CI [0.50–0.80]) and stroke (HR 0.86 [0.54–1.35] (Fig. 2).

### Subgroup and sensitivity analysis

**Fig. 3:**
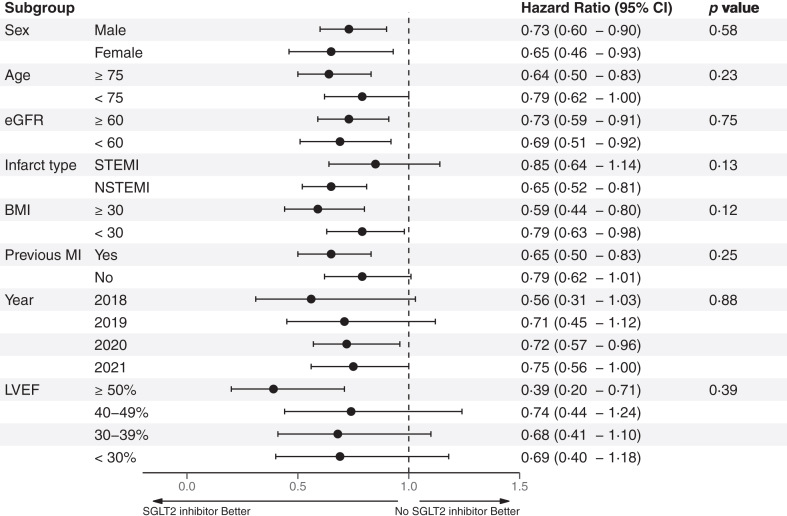
Forest plot of the subgroup analysis of the primary outcome.

The results of the subgroup analysis are presented in a Forest plot demonstrating consistently lower hazard ratios for the primary outcome in those treated with an SGLT2 inhibitor among all subgroups with no significant interactions (Fig. 3). The results of the complete-case sensitivity analyses of the composite outcome (HR 0.67; 95% CI [0.51–0.88]) and first hospitalisation for heart failure (HR 0.66; 95% CI [0.46–0.95]) were consistent with the main analysis with lower hazard ratios among SGLT2 inhibitor-treated patients (Fig. 2). The results of the main analysis model were consistent when adding invasive coronary angiography and PCI during hospitalisation and angiographic findings (Fig. 2). The complete-case analysis of all-cause death (HR 0.70; 95% CI [0.47–1.04]) and stroke (HR 0.66 [0.29–1.50]) were numerically lower but were not statistically significant. The inverse probability-weighted sensitivity analysis was consistent with the primary analysis for the composite outcome (HR 0.61 [0.49–0.76]) and all-cause death (HR 0.49 [0.36–0.66]) while the analysis of first hospitalisations for heart failure (HR 0.76 [0.55–1.03]) or first stroke (HR 0.76 [0.42–1.38]) were not statistically significant. The sensitivity analysis of patients with a low risk of heart failure demonstrated a significantly lower risk for the primary outcome and all-cause death (Supplemental Table S2).

## Discussion

In this observational study in patients with type 2 diabetes with acute myocardial infarction, we found that SGTL2 inhibitor treatment was associated with lower rates of first hospitalisation for heart failure and all-cause death. During the one year of follow-up, treatment with SGLT2 inhibitors was associated with a 30% lowered hazard ratio of the primary composite outcome of first hospitalisation for heart failure or all-cause death. This finding was consistent in the sensitivity analyses and among all subgroups.

The hazard ratios for reduction of hospitalisations for heart failure (0.78) are similar to the findings in DAPA-MI (0.83) and EMPACT-MI (0.77). This is in agreement with the findings from a meta-analysis of the five major cardiovascular outcome trials which found a 30% mean reduction of hospitalisations for heart failure with SGLT2 inhibitor therapy among those with established cardiovascular disease.^15^ However, our finding of a significant reduction in mortality could not be demonstrated in either DAPA-MI or EMPACT-MI. This could potentially be explained by different baseline demographics where we had a generally older and sicker population in our real-world registry population and all our patients had type 2 diabetes while previous diabetes was an exclusion criterion in DAPA-MI and type 2 diabetes patients only constituted about a third of the EMPACT-MI population. Both trials excluded patients with a diagnosis of previous heart failure while we had about 12% previous heart failure patients. Furthermore, our finding of reduced mortality is in line with a meta-analysis of the cardiovascular outcome trials of SGLT2 inhibitors in patients with type 2 diabetes and established cardiovascular disease demonstrating a significant reduction in mortality.^15^ Patients discharged with SGLT2 inhibitors presented more often with ST-elevation myocardial infarction and had worse LVEF at index hospitalisation but despite this, patients on SGLT2 inhibitors had lower rates of first hospitalisation for heart failure and lower mortality than patients not treated with SGLT2 inhibitors. The improved outcomes were seemingly robust for all levels of LVEF and regardless of myocardial infarction classification (NSTEMI or STEMI) as seen in Fig. 3 with no significant subgroup interactions. A more detailed analysis stratified by classification of myocardial infarction showed similar results among the majority of subgroups (Supplemental Figure S2). A trend towards little to no benefit on all-cause death among patients with NSTEMI was observed which possibly could be due to higher rates of non-cardiac mortality. The improved outcomes were also confirmed in the sensitivity analysis of patients with a low risk of heart failure (Supplemental Table S2). However, the analyses should be interpreted cautiously as confidence intervals were wide and the study was not powered for these analyses.

The specific mechanisms of how SGLT2 inhibitors reduce the risk of cardiovascular events have still not been completely elucidated, although several mechanisms have been proposed, including effects on cardiomyocyte metabolism and anti-inflammatory mechanisms.^16^ In the acute post-myocardial infarction setting, SGLT2 inhibitor treatment has been associated with smaller infarct size, lower in-hospital arrhythmias and in-hospital cardiovascular mortality, and reduced levels of NT-proBNP suggesting acute effects.^17^, ^18^, ^19^ Intensive glucose-lowering therapy has not clearly correlated with lower rates of cardiovascular events or lowered mortality, and the cardioprotective effect of SGLT2 inhibitors does not appear to be related to the glucose-lowering effect.^20^^,^^21^ Interestingly, the association between SGLT2 inhibitor treatment and lowered event rates remained consistent in patients with estimated GFR below 60 mL/1.73 m^2^. Treatment with SGLT2 inhibitors appears to be cardioprotective regardless of indication and effective even among those with deteriorating eGFR.^22^^,^^23^

One of the main messages from DAPA-MI and EMPACT-MI trials was that it was safe to prescribe dapagliflozin in the early post-myocardial infarction phase in patients without established diabetes or congestive heart failure. Our study indicates that it is efficacious to start treatment with an SGLT2 inhibitor in the acute and subacute phase of myocardial infarction but should be interpreted in the context of the observational design using all patients in one country in contrast to the randomised clinical trials enrolling highly selected patients.^11^^,^^24^

By design, this study has limitations. Due to the observational nature of this study, we cannot disregard any bias that might have affected exposure or outcomes, such as treatment bias, drug adherence, or differences in patient characteristics. Additionally, we did not examine in-hospital adverse events or in-hospital mortality and did not differentiate between different types of SGLT2 inhibitors. We only included patients with an eGFR >30 mainly because SGLT2 inhibitor treatment was contraindicated in eGFR ≤30 for the majority of the study period and therefore seldom used for patients with low GFR. LVEF and eGFR were obtained during the index hospitalisation. One of the main strengths of this study is that we included patients from nationwide registries with information on prescribed medications, diagnosis and mortality registries with close to complete coverage.

In conclusion, in patients with type 2 diabetes, treatment with SGLT2 inhibitors after myocardial infarction was associated with lower rates of all-cause mortality and first hospitalisation for heart failure when compared to patients treated with standard of care.

## Contributors

D.E., M.A.M. and H.C.R. designed the study. H.C.R. wrote the first draft and later revisions of the manuscript. M.A.M. and H.C.R. conducted the statistical analyses. H.C.R., M.A.M, D.E., T.J., S.J., and J.O. contributed to the discussion and reviewed and edited the manuscript. D.E. is the guarantor of this work and, as such, had full access to all data used in the study and takes responsibility for the integrity of the data and the accuracy of data analysis.

## Data sharing statement

Data from SWEDEHEART is legally restricted and not publicly available. Aggregated data can be granted upon after requests through the Uppsala Clinical Research Center (UCR).

## Declaration of interests

Jonas Oldgren reports institutional grants from AstraZeneca for projects unrelated to this study. Stefan James reports institutional grants from AstraZeneca, Novartis, Amgen and Jansen for projects unrelated to this study and participation on advisory boards for studies conducted by New Amsterdam Pharma. David Erlinge reports participation on advisory boards for AstraZeneca. The rest of the co-authors report no conflicts of interest.
