# Supplementary material for: SGLT2 inhibitors for patients with type 2 diabetes mellitus after myocardial infarction: a nationwide observation registry study from SWEDEHEART
Source: Lancet Reg Health Eur. 2024 Aug 21;45:101032. doi: 10.1016/j.lanepe.2024.101032 (PMC11387207; doi:10.1016/j.lanepe.2024.101032)
Supplement: Supplemental Tables and Figures [file mmc1.pdf]

Supplementary Material

Manuscript: SGLT2 inhibitors for patients with type 2 diabetes mellitus after myocardial infarction: a nationwide observation registry study from SWEDEHEART

Table of Contents

Supplemental Table 1. Procedural data from coronary angiographies.	Page 2
Supplemental Table 2. Results of the primary and secondary outcome analyses with one-year follow-up in a population with a low risk of heart failure.	Page 3
Supplemental Figure 1. Flowchart of the number of patients in the analysis after inclusion and exclusion criteria were applied.	Page 4
Supplemental Figure 2. Results of the primary and secondary outcome analyses with one-year follow-up in a population stratified by myocardial infarction classification.	Page 5

Supplemental Table 1.

Procedural data from coronary angiographies.

	SGLT2 inhibitor	No SGLT2 inhibitor	Missing	p-value
N	2498 (22·2%)	8773 (77·8%)	–	–
Coronary angiography	2411 (96·5%)	7905 (90·1%)	0·0%	<0·001
PCI*	2074 (83·0%)	6532 (74·5%)	0·0%	<0·001
Number of vessels diseased*			9·0%	0·086
1VD	883 (35·4%)	2697 (30·7%)		
2VD	600 (24·0%)	2038 (23·2%)		
3VD	583 (23·3%)	1859 (21·2%)		
Any LM-disease	208 (8·3%)	790 (9·0%)		
Proximal disease*	1413 (56·6%)	4698 (53·6%)	9·0%	0·386
Bifurcation PCI*	280 (11·2%)	847 (9·7%)	9·0%	0·232

PCI = percutaneous coronary intervention; VD = vessel disease. *Percentages were calculated using the total study population as the denominator.

Supplemental Table 2.

Results of the primary and secondary outcome analyses with one-year follow-up in a population with a low risk of heart failure.

	Unadjusted HR (95% CI)	Adjusted HR (95% CI)
Composite of first hospitalisation for heart failure or all-cause death	0·39 (0·26–0·60)	0·58 (0·37–0·91)
First hospitalisation for heart failure	0·50 (0·27–0·93)	0·66 (0·34–1·28)
All-cause death	0·36 (0·21–0·63)	0·54 (0·31–0·96)

Adjusted for age, sex, eGFR, BMI, HbA1c-level, smoking status, clinical frailty score, Killip class at admission, MI classification (STEMI/NSTEMI), left ventricular ejection fraction, year of admission, treatment with angiotensin-converting enzyme inhibitors/ angiotensin II receptor blockers, beta-blockers, statins, aspirin and insulin, diagnosis of hypertension and COPD, and prior stroke, heart failure, and myocardial infarction. CI = Confidence interval; HR = Hazard ratio; KM = Kaplan-Meier.

Supplemental Figure 1.

Flowchart of the number of patients in the analysis after inclusion and exclusion criteria were applied.

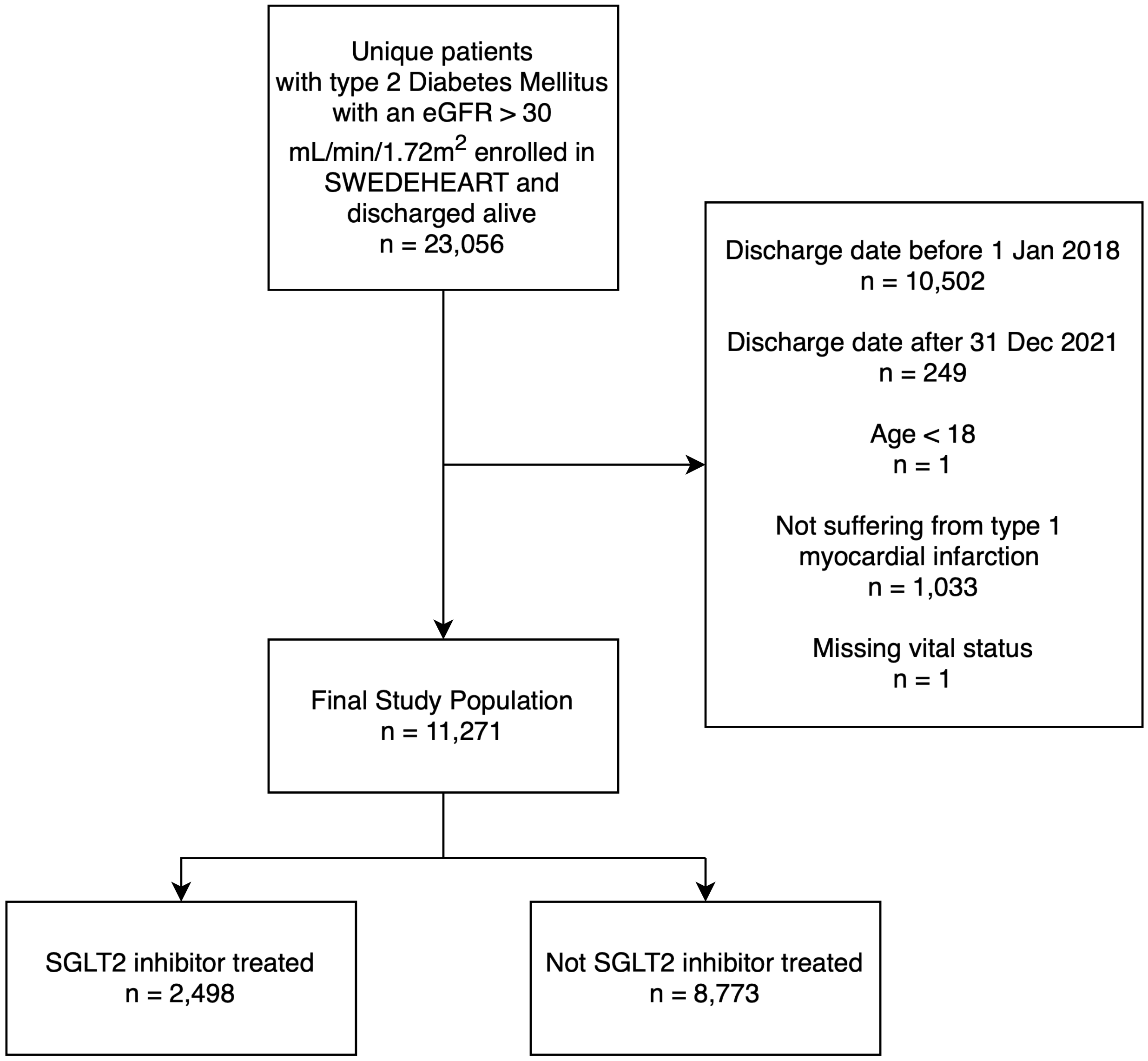

eGFR = estimated glomerular filtration rate; SGLT2 = Sodium-glucose co-transporter 2; SWEDEHEART = Swedish Web-system for Enhancement and Development of Evidence-based care in Heart disease Evaluated According to Recommended Therapies.

Supplemental Figure 2.
Results of the primary and secondary outcome analyses with one-year follow-up in a population stratified by myocardial infarction classification.

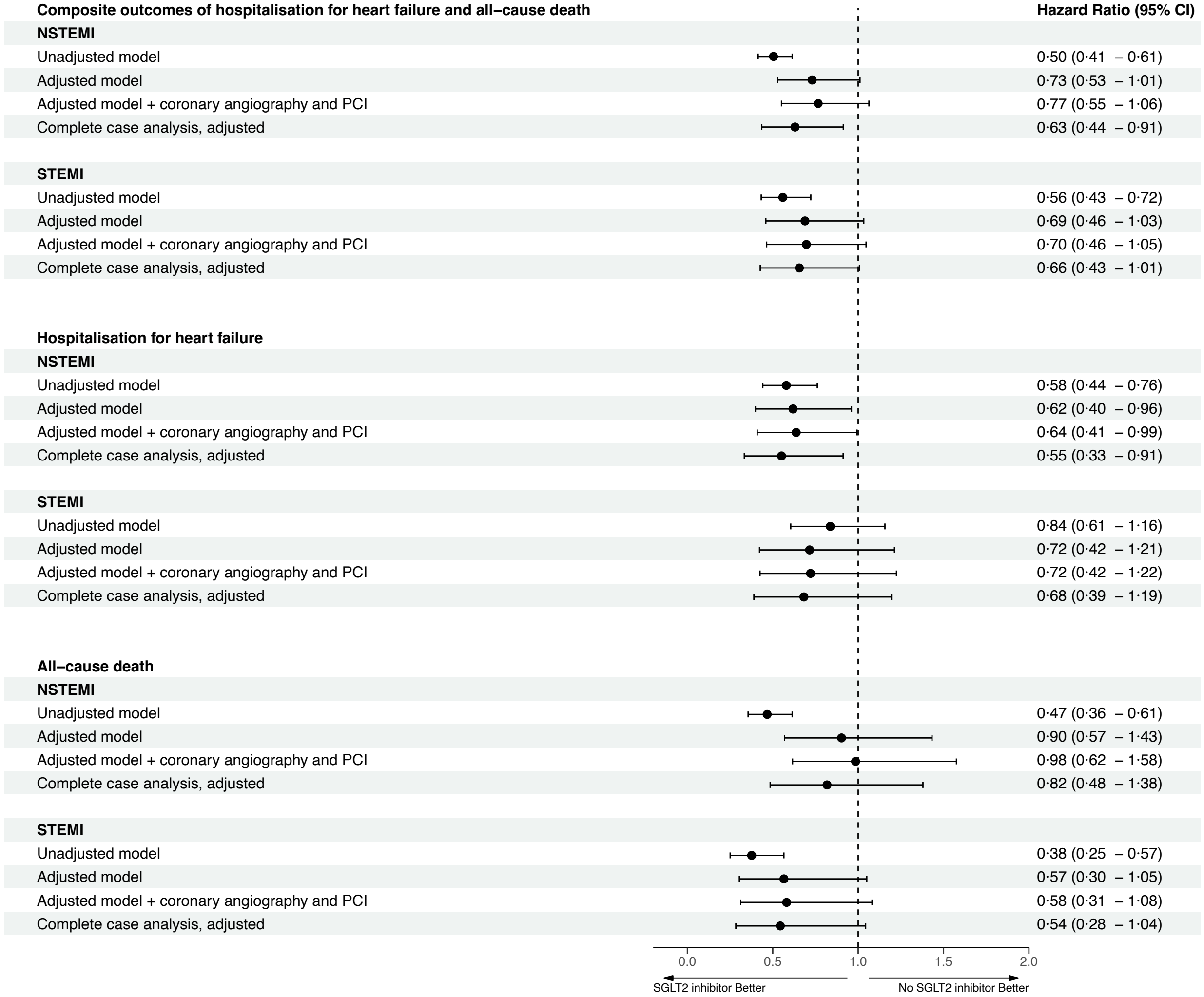

Adjusted for age, sex, eGFR, BMI, HbA1c-level, smoking status, clinical frailty score, Killip class at admission, left ventricular ejection fraction, year of admission, treatment with angiotensin-converting enzyme inhibitors/ angiotensin II receptor blockers, beta-blockers, statins, aspirin and insulin, diagnosis of hypertension and COPD, and prior stroke, heart failure, and myocardial infarction.
Additional adjustment for invasive coronary angiography and PCI during index hospitalisation were added to the specified model.
CI = Confidence interval; HR = hazard ratio; NSTEMI = non-ST-elevation myocardial infarction; PCI = percutaneous coronary intervention; SGLT2 = Sodium-glucose co-transporter 2; STEMI = ST-elevation myocardial infarction.
